# What are Risk Factors of Postoperative Pneumonia in Geriatric Individuals after Hip Fracture Surgery: A Systematic Review and Meta‐Analysis

**DOI:** 10.1111/os.13631

**Published:** 2022-12-15

**Authors:** Yu‐Cheng Gao, Yuan‐Wei Zhang, Liu Shi, Wang Gao, Ying‐Juan Li, Hui Chen, Yun‐Feng Rui

**Affiliations:** ^1^ Department of Orthopaedics, Zhongda Hospital, School of Medicine Southeast University Nanjing China; ^2^ Multidisciplinary Team (MDT) for Geriatric Hip Fracture Management, Zhongda Hospital School of Medicine, Southeast University Nanjing China; ^3^ School of Medicine Southeast University Nanjing China; ^4^ Orthopaedic Trauma Institute (OTI) Southeast University Nanjing China; ^5^ Trauma Center, Zhongda Hospital, School of Medicine Southeast University Nanjing China; ^6^ Department of Geriatrics, Zhongda Hospital, School of Medicine Southeast University Nanjing China

**Keywords:** Elderly, Hip Fracture, Incidence, Postoperative Pneumonia, Risk Factors

## Abstract

Postoperative pneumonia (POP) is a common postoperative complication. Negative consequences associated with POP included prolonged hospital length of stay, more frequent intensive care unit (ICU) stays, and a higher rate of sepsis, readmission, and mortality. This meta‐analysis aimed to assess the incidence and risk factors associated with POP after hip fracture surgery in elderly patients. PubMed, Web of Science, and Cochrane Library were searched (up to March 31, 2022). All studies on the risk factors for POP after hip fracture surgery in elderly patients, published in English, were reviewed. The qualities of the included studies were assessed using the Newcastle–Ottawa Scale. Data were pooled, and a meta‐analysis was performed. Ten studies, including 12,084 geriatric patients undergoing hip fracture surgery, were included. Of these 12,084 patients, POP occurred in 809 patients. The results indicated that age (mean difference [*MD*] = 4.95, 95% confidence interval [*CI*]: 3.22–6.69), male (odds ratio [*OR*] = 1.41, 95% *CI*: 1.02–1.93), the American Society of Anaesthesiologists classification ≥3 (*OR* = 3.48, 95% *CI*: 1.87–6.47), dependent functional status (*OR* = 5.23, 95% *CI*: 2.18–12.54, *P* = 0.0002), smoking (*OR* = 1.33, 95% *CI*: 1.07–1.65), chronic obstructive pulmonary disease (*OR* = 3.76, 95% *CI*: 2.07–6.81), diabetes mellitus (*OR* = 1.19, 95% *CI*: 1.01–1.40), coronary heart disease (*OR* = 1.74, 95% *CI*: 1.23–2.46), arrhythmia (*OR* = 1.47, 95% *CI*: 1.01–2.14), cerebrovascular disease (*OR* = 1.88, 95% *CI*: 1.56–2.27), dementia (*OR* = 2.36, 95% *CI*: 1.04–5.36), chronic renal failure (*OR* = 1.85, 95% *CI*: 1.29–2.67), hip arthroplasty (*OR* = 1.30, 95% *CI*: 1.08–1.56), delayed surgery (*OR* = 6.40, 95% *CI*: 3.00–13.68), preoperative creatinine (*MD* = 5.32, 95% *CI*: 0.55–10.08), and preoperative serum albumin (*MD* = −3.01, 95% *CI*: −4.21 – −1.80) were risk factors for POP. Related prophylactic measures should be provided in geriatric patients with the above‐mentioned risk factors to prevent POP after hip fracture surgery.

## Graphical abstract

We found that age, gender, ASA classification ≥ 3, dependent function status, smoking, COPD, diabetes mellitus, coronary heart disease, arrhythmia, cerebrovascular disease, dementia, chronic renal failure, hip arthroplasty, delayed surgery, preoperative creatinine, preoperative serum albumin were the risk factors of POP *via* meta‐analysis of 10 included studies.

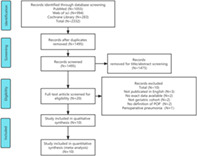

## Introduction

Morbidity in geriatric hip fractures is increasing with an increase in the aging population worldwide.^1^ Early surgery is recommended for such patients.^2^ Postoperative pneumonia (POP) is the commonest postoperative complication, with an incidence of 3.5%–15.2%, ranking second to delirium.^3^, ^4^, ^5^, ^6^, ^7^, ^8^, ^9^, ^10^, ^11^, ^12^, ^13^ Negative outcomes associated with POP included prolonged hospital length of stay, more frequent intensive care unit (ICU) stays, and a higher rate of sepsis, readmission, and mortality.^14^, ^15^, ^16^ The 30‐day mortality rate for elderly patients who develop POP after hip fracture surgery was 27%–43%.^7^, ^15^ It is crucial to have appropriate awareness of population‐specific risk factors, given the high prevalence and associated poor outcomes of POP, to identify susceptible patients and implement preventative measures.^17^

Several studies on the risk factors for POP in elderly patients with hip fractures exist. However, these studies had some limitations such as ambiguous description of targeted population, non‐uniform or unclear diagnostic criteria for POP sweeping statement of factors, relative limited sample size, and so on, which led to inconsistent or even conflicting results. It is still unclear if these risk factors from individual studies can predict POP after hip fracture surgery in elderly patients because of discrepancies in study design, sample size, and the level of evidence, which might affect clinical judgment and treatment. This meta‐analysis aimed to (i) evaluate the quality of existing studies and select suitable studies for meta‐analysis to resolve the inconsistency caused by the limitations in a single study; (ii) come to a relatively accurate conclusion on the risk factors for POP in elderly patients undergoing hip fracture surgery and evaluate the level of evidence on the pooled results of each factor; (iii) provide evidence for clinical decision‐making and preventive strategies.

## Methods

Two reviewers searched PubMed, Web of Science, and Cochrane Library from inception until March 31, 2022. A combined search strategy of keywords and random words was adopted. The keywords “hip fracture,” “femoral neck fracture,” “intertrochanteric fracture,” “per trochanteric fracture,” “hip surgery,” “postoperative pneumonia,” and “POP” were used in combination. The concrete search strategy for PubMed was as follows ([hip fracture] OR [intertrochanteric fracture] OR [per trochanteric fracture] OR [femoral neck fracture] OR [hip surgery] AND [postoperative pneumonia] OR [POP]). Only studies published in English were included, and the reference lists of all eligible studies and relevant reviews were manually searched for additional studies.

### Eligibility Criteria

The inclusion criteria were as follows: (1) participants aged over 65 years undergoing any surgical intervention for a hip fracture; (2) original studies, including prospective or retrospective cohort studies and other designs where a validated diagnostic criterion was used to determine the presence/absence of POP; (3) participants who were divided into two groups (POP and non‐POP or similar group name) according to the presence/absence of POP for comparison and identification of the associated risk factors; (4) number of patients with and without POP and at least one set of data on their clinical characteristics.

The exclusion criteria were as follows: (1) cases described as “chest infection,” which is not precisely the same as pneumonia; (2) fracture type described as proximal femur fracture or other inexact statements; (3) mixed sample studies wherein the data for different operative types are not independently presented; (4) studies including patients undergoing hip surgery for osteoarthritis or other non‐fracture causes; (5) studies including patients who experienced multiple fractures or pathological fractures.

### Study Identification

Two reviewers independently scanned the titles and/or abstracts of potentially included studies. The same two reviewers thoroughly reviewed the full texts of the studies deemed potentially eligible. Full texts considered eligible by both two reviewers were included. Disagreements on study eligibility were resolved by discussion until a consensus was reached or by consulting another senior reviewer until a consensus was reached.

### Data Extraction

Two reviewers independently extracted data concerning the studies, patient characteristics, outcomes, and follow‐up.

#### Extracted Data

##### Study Variables

The study variables were as follows: country of study origin, study design, target cohort, the time interval for study conduction, the number of all participants, and the number of participants with or without POP.

##### Participant Variables

The following were the participant variables: age; sex; body mass index; American Society of Anaesthesiologists (ASA) classification; fracture type; functional status; history of smoking; comorbidities, including chronic obstructive pulmonary disease (COPD), diabetes mullites (DM), hypertension, coronary heart disease (CHD), chronic heart failure (CHF), arrhythmia, cerebrovascular disease (CVA), dementia, chronic renal failure (CRF), and cancer.

##### Intraoperative Variables

The intraoperative variables were as follows: anesthetic type, surgical procedure, surgical duration, and intraoperative blood loss.

##### Other Perioperative Variables

The other perioperative variables were as follows: time from injury to surgery; delayed surgery; preoperative hemoglobin, creatinine (Cr), serum albumin (Alb), and hypoalbuminemia.

#### Quality Assessment

The Newcastle–Ottawa Scale (NOS) was used to assess the quality of the evidence.^18^ Two reviewers independently undertook critical appraisal. Disagreements were resolved *via* discussion or consulting a third reviewer until a consensus was reached. Each study was scored out of a maximum of nine points. A study with a score of 0‐3 points, 4‐6 points, and 7‐9 points was regarded as a low‐quality (LQ) study, a moderate‐quality (MQ) study, and a high‐quality (HQ) study, respectively.^19^

### Statistical Analysis

The study aimed to determine the factors associated with POP in geriatric patients with hip fractures. Review Manager 5.3 software (Cochrane Collaboration, Oxford, UK) was used to conduct the meta‐analysis. For dichotomous data, the risk ratio or odds ratio (*OR*) with a 95% confidence interval (*CI*) was calculated, and for continuous outcomes, the mean difference (*MD*) with the standard deviation (*SD*) was calculated. For all analyses, 95% *CI*s and forest plots were calculated. *P*‐value and I‐value according to the standard chi‐square test were used to assess statistical heterogeneity. No significant heterogeneity existed among these studies if *I* ^2^ < 50% or *P* > 0.1. A fixed effects model was then applied for the meta‐analysis, or else a random effects model was used.

### Evidence Level

Evidence was considered strong if it was derived from at least three studies, including two or more HQ studies, which were statistically homogeneous. Evidence was considered weak if it was derived from at least three MQ or LQ studies that were statistically heterogeneous. Evidence derived from two studies was considered limited, or else the evidence was considered moderate. Evidence from a single study was not evaluated. If a positive result was obtained from studies with significant heterogeneity and could not be explained by sensitivity analysis, or if the result was altered or reversed by excluding one of the included studies, we would consider that this result was unreliable and that this factor was not suitable for the meta‐analysis.

## Results

### Search Results

**Figure 1:**
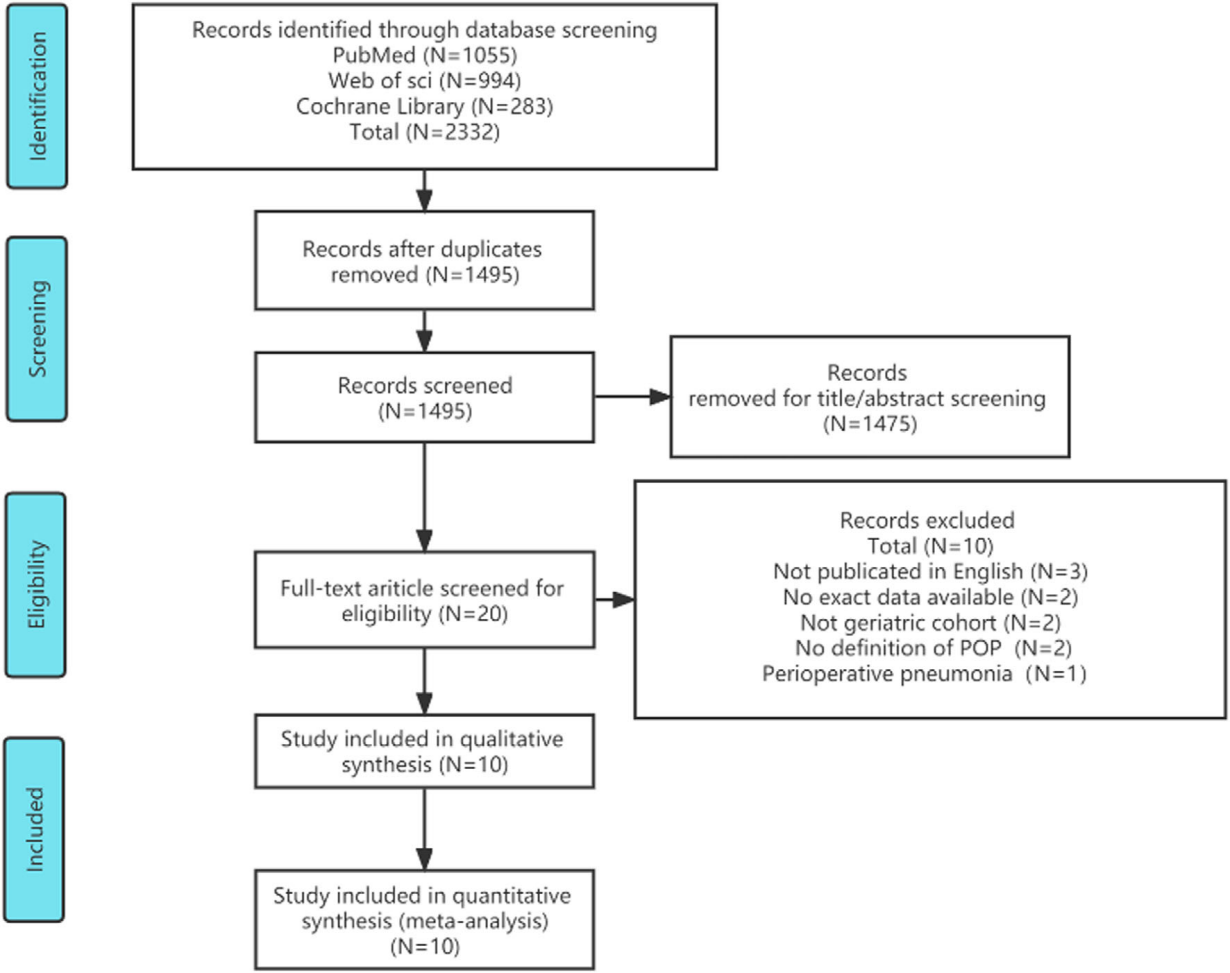
PRISMA flowchart of the search strategy results

A total of 2332 studies were identified from the databases. After eliminating the duplicates, 1495 studies remained. Following this, the titles and abstracts were scanned to eliminate studies which did not meet the inclusion criteria. Consequently, 1475 citations were excluded. Following this, the full texts of the remaining 20 studies were carefully reviewed to identify those that met the inclusion criteria. Finally, 10 original articles involving 12,084 patients published between 2016 and 2022 were included in this meta‐analysis (Fig. 1).

### The General Characteristic of the Included Studies

Nine case‐control studies^5^, ^6^, ^7^, ^8^, ^9^, ^10^, ^11^, ^12^, ^13^, ^20^ and one prospective cohort study^4^ involving 12,084 patients were included in the meta‐analysis, and 6.7% (809) of the patients developed POP. Seven of the 10 included studies were conducted in China. In nine of the 10 studies,^4^, ^5^, ^6^, ^8^, ^9^, ^10^, ^11^, ^12^, ^13^ the average or median age of the participants was >70 years. In the one other study, the patient distribution across different age groups were reported rather than the average age; however, age >65 years was one of the inclusion criteria. The male: female ratios of the non‐POP group in all studies and that of the POP group in eight out of 10 studies were <1.0. The male: female ratios in three studies^4^, ^8^, ^12^ were lower in the POP group than in the non‐POP group. The fracture type of the included studies was restricted to hip fractures, namely femoral neck fractures, intertrochanteric fractures, or per trochanteric fractures, and was analyzed as a risk factor. One study included one or more fracture types. The surgical procedure varied with the fracture type and mainly comprised hip arthroplasty and internal fixation. Total hip arthroplasty (THA) or hip hemiarthroplasty (HHA) was the most common surgical procedure for femoral neck fractures. Intramedullary fixation was preferred for intertrochanteric fractures. According to the NOS, most studies were MQ or HQ studies, with a score ranging from 5 to 7 (Table 1). POP occurred before discharge in eight out of 10 studies, while two other studies^9^, ^12^ conducted a 30‐day follow‐up. Precise and similar diagnostic criteria for POP were represented, and the presence of pneumonia preoperatively or at admission was one of the exclusion criteria in all the included studies. Our study has language bias because only studies published in English were included.

**Table 1:** Detailed information on the basic characteristics of the 10 included studies and participants

Study (published year)	Period	Country	Sample size (P/N)	Mean age (P/N)	Male/female ratio (P/N)	Fracture type	Surgical procedure	Significant risk factors in conclusion	NOS	Quality
Salarbaks^10^ (2020)	2015.1–2016.12	Netherlands	62/345	83.1/83.4	0.88/0.40	Intracapsular fracture Extracapsular fracture	HHA PFNA	Male gender; COPD	6☆	MQ
Xiang^21^ (2020)	2014.8–2019.10	China	166/947	86.4/78.8	0.47/0.54	Intertrochanteric fracture	NM	BMI; CRP; preoperative serum albumin; functional status; time from injury to surgery	6☆	MQ
Lv^4^ (2016)	2001.1–2012.12	China	70/1395	83.1/73.9	0.46/0.73	Femoral neck fracture Intertrochanteric fracture	Intramedullary fixation Hip replacement Other procedures	Age; anemia; DM; prior stroke; number of comorbidities; ASA; Hypoproteinemia; surgical type; serum Cr; RDW; mechanical ventilation	6☆	MQ
Shin^5^ (2020)	2010.1–2019.10	Korea	59/1096	83.1/77.9	0.55/0.37	Hip fracture	Osteosynthesis Arthroplasty	Age; CVD; early postoperative hypoalbuminemia	7☆	HQ
Zhao^7^ (2020)	2014.10–2018.12	China	53/1442	NM	1.12/0.40	Hip fracture	Implant Intramedullary devices Extramedullary devices	Age > 82 years; chronic respiratory disease; liver disease; urinary tract infection; CKMB >20 U/l; BNP ≥75 ng/l; D‐dimer >2.26 mg/l	7☆	HQ
Byun^13^ (2018)	2015.1–2018.2	Korea	38/394	83.7/78.6	0.52/0.40	Hip fracture	NM	Longer duration of surgery; delayed surgery; age; low BMI and malnutrition (hypoalbuminemia)	7☆	HQ
Zhang^11^ (2022)	2010.4–2018.4	China	70/1215	82.6/79.4	0.75/0.42	Hip fracture	THA HHA Intramedullary fixation multiple screw plate/screw	COPD; number of comorbidities; ASA classification > 2; preoperative dependent functional status; cognitive impairment	7☆	HQ
Ji^9^ (2021)	2018.1–2019.11	China	55/846	81.6/78.5	0.72/0.50	Femoral neck fracture Intertrochanteric fracture	THA HHA Intramedullary fixation	COPD; PaO_2_ and hypoxemia (PaO_2_ <72.5 mmHg); time from injury to surgery	7☆	HQ
Tian^6^ (2022)	2016.1–2020.12	China	182/2965	79.3/75.7	1.14/0.46	Hip fracture	Osteosynthesis Arthroplasty	Age; male gender; respiratory disease; heart disease; CVA; liver disease; preoperative stay; general anesthesia	5☆	MQ
Wang^12^ (2019)	2018.1–2018.12	China	54/666	82.3/77.5	0.59/0.69	Femoral neck fracture	THA HHA Intramedullary fixation	Hypoalbuminemia; COPD; prior stroke; time from injury to surgery	6☆	MQ

Abbreviations: ASA, American Society of Anesthesiologist classification; COPD, chronic obstructive pulmonary disease; Cr, creatinine; CVA, cerebrovascular accident; CVD, cardiovascular disease; DM, diabetes mellitus; HHA, hip hemiarthroplasty or half hip arthroplasty; N, non‐POP group; NM, not mentioned; P, pneumonia group; PaO_2_, arterial pressure of oxygen; PFNA, proximal femoral nail antirotation; RDW, red blood cell distribution width; THA, total hip arthroplasty.

### Meta‐Analysis

The results of the meta‐analysis indicated that age (*MD* = 4.95, 95% *CI*: 3.22–6.69), male (*OR* = 1.41, 95%*CI*: 1.02–1.93), ASA classification ≥3 (*OR* = 3.48, 95% *CI*: 1.87–6.47), dependent functional status (*OR* = 5.23, 95% *CI*: 2.18–12.54), smoking (*OR* = 1.33, 95% *CI*: 1.07–1.65), COPD (*OR* = 3.76, 95% *CI*: 2.07–6.81), DM (*OR* = 1.19, 95% *CI*: 1.01–1.40), CHD (*OR* = 1.74, 95% *CI*: 1.23–2.46), arrhythmia (*OR* = 1.47, 95% *CI*: 1.01–2.14), CVA (*OR* = 1.88, 95% *CI*: 1.56–2.27), dementia (*OR* = 2.36, 95% *CI*: 1.04–5.36), CRF (*OR* = 1.85, 95% *CI*: 1.29–2.67), hip arthroplasty (*OR* = 1.30, 95% *CI*: 1.08–1.56), delayed surgery (*OR* = 6.40, 95% *CI*: 3.00–13.68), preoperative Cr (*MD* = 5.32, 95% *CI*: 0.55–10.08), and preoperative Alb (*MD* = ‐3.01, 95% *CI*: −4.21 – −1.80) were the risk factors for POP in geriatric individuals after hip fracture surgery.

### Demographic Variables‐Related Risk Factors

#### Age

**Figure 2:**
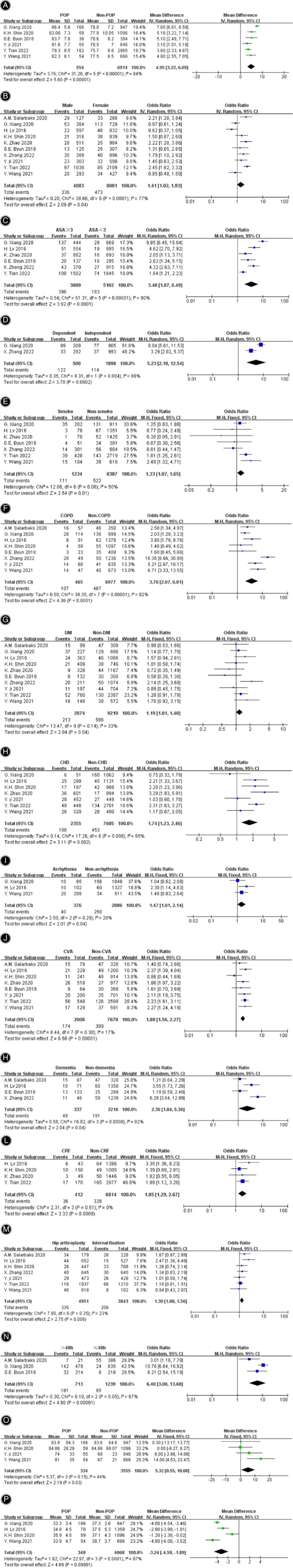
The forest plots of the meta‐analyses of certain variables comparing the characteristics of elderly hip fracture patients with and without postoperative pneumonia (POP). Elderly patients with the risk factors of (A) age, (B) male sex, (C) American Society of Anaesthesiologists classification ≥3, (D) dependent functional status, (E) history of smoking, (F) chronic obstructive pulmonary disease, (G) diabetes mellitus, (H) coronary heart disease, (I) arrhythmia, (J) cerebrovascular disease, (K) dementia, (L) chronic renal failure, (M) hip arthroplasty, (N) delayed surgery (time from injury to surgery of >48 h), (O) preoperative creatinine, and (P) preoperative albumin would be more likely to develop pneumonia after hip fracture surgery. The width of the horizontal line represents the 95% confidence interval (CI) of the individual studies, and the square proportion represents the weight of each study. The diamond represents the pooled odds ratio or mean difference and 95% CI.

In six studies^5^, ^6^, ^8^, ^9^, ^12^, ^13^ involving 7486 patients, the age was presented as the *mean* ± *SD* for each group. A random effects model was applied because significant heterogeneity was observed among these studies (*I* ^2^ = 84%). A significant difference was observed in terms of the age between patients with and without POP (*MD* = 4.95, 95% *CI*: 3.22–6.69, *P* < 0.0001) (Table 2, Fig. 2a). A subgroup analysis was attempted based on nationality, mean age, and fracture type; however, significant heterogeneity was not ruled out. Heterogeneity might be the result of the multiple factors involved. However, after eliminating three MQ studies,^6^, ^8^, ^12^ the *I* ^2^ value for three HQ studies^5^, ^9^, ^13^ decreased to 16%, and the significance remained unchanged (*MD* = 4.41, 95% *CI*: 3.16–5.67, *P* < 0.0001), indicating the reliability of the result (Fig. S1a).

**TABLE 2:** Detailed data on potential risk factors for postoperative pneumonia (POP) following hip fracture surgery and the results of meta‐analysis

					Statistical heterogeneity		
Variables	*N* (Study number)	Model	*OR* or *MD* (95%CI)	*p*	*I* ^2^ (%)*	*Chi* ^2^	Evidence level
Age	7486 (6)	Random	4.95 (3.22, 6.69)	<0.00001	84	31.28	Moderate
Male sex	12,084 (10)	Random	1.41 (1.02, 1.93)	<0.0001	77	38.66	Moderate
BMI	5750(6)	Random	−0.31 (−0.89, 0.27)	0.29	75	20.13	Moderate
ASA ≥ 3	8911 (6)	Random	3.48 (1.87, 6.47)	<0.0001	90	51.31	Moderate
Fracture type	7169 (5)	Random	0.96 (0.69, 1.35)	0.83	62	10.54	Moderate
Dependent function status	2398 (2)	Random	5.23 (2.18, 12.54)	0.0002	88	8.31	Limited
Smoke	9621 (7)	Fixed	1.33 (1.07, 1.65)	0.01	50	12.06	Strong
COPD	7442 (8)	Random	3.12 (1.82, 5.36)	<0.0001	86	51.33	Moderate
DM	12,084 (10)	Fixed	1.19 (1.01, 1.40)	0.04	33	13.47	Strong
Hypertension	11,425 (8)	Random	1.02 (0.71, 1.46)	0.91	78	31.62	Moderate
CHD	9960 (7)	Random	1.74 (1.23, 2.46)	0.002	65	17.26	Moderate
CHF	1520 (2)	Fixed	0.95 (0.53, 1.71)	0.86	0	0.12	Limited
Arrhythmia	3262 (3)	Fixed	1.47 (1.01, 2.14)	0.04	20	2.5	Moderate
CVA	7686 (8)	Fixed	1.88 (1.56, 2.27)	<0.0001	17	8.44	Strong
Dementia	3553 (4)	Random	2.36 (1.04, 5.36)	0.04	82	16.82	Moderate
CRF	7226 (4)	Fixed	1.85 (1.29, 2.67)	0.0009	0	2.31	Strong
Cancer	7616 (5)	Fixed	1.33 (0.87, 2.03)	0.19	8	4.33	Strong
Anesthesia type	7879 (6)	Random	1.11 (0.81, 1.53)	0.50	58	11.89	Moderate
Hip arthroplasty	8794 (7)	Fixed	1.30 (1.08, 1.56)	0.006	23	7.80	Strong
Surgical duration	7808 (6)	Random	2.09 (−3.13, 7.30)	0.43	74	19.04	Moderate
Intra‐operative blood loss	6263 (4)	Fixed	3.96 (−16.27, 24.19)	0.70	0	0.70	Strong
Time from injury to surgery	7418 (5)	Random	2.84 (0.94, 4.74)	0.003	89	37.72	Unreliable
Delayed surgery^†^	1952 (3)	Random	6.40 (3.00, 13.68)	<0.0001	67	6.10	Moderate
Preoperative Hb	4273 (4)	Random	−3.11 (−7.21, 0.99)	0.14	82	16.84	Moderate
Preoperative Alb	4417 (4)	Random	−3.24 (−4.59, −1.89)	<0.0001	87	22.97	Moderate
Preoperative Cr	3889 (4)	Fixed	5.32 (0.55, 10.08)	0.03	44	5.37	Strong
Preoperative hypoalbuminemia	3932 (4)	Random	3.33 (1.32, 8.37)	0.01	90	29.04	Unreliable

Abbreviations: Alb, albumin; ASA, American Society of Anesthesiologist classification; BMI, body mass index; COPD, chronic obstructive pulmonary disease; CHD, coronary heart disease; CHF, chronic heart failure; Cr, creatinine; CRF, chronic renal failure; CVA, cerebrovascular accident; DM, diabetes mellitus; Hb, hemoglobin; *MD*, mean difference; *OR*, odd ratio.

* *I* ^2^ statistic was defined as the proportion of heterogeneity not due to chance or random error.

^†^ Defined as time from injury to surgery more than 48 h.

#### Sex

**Figure 3:**
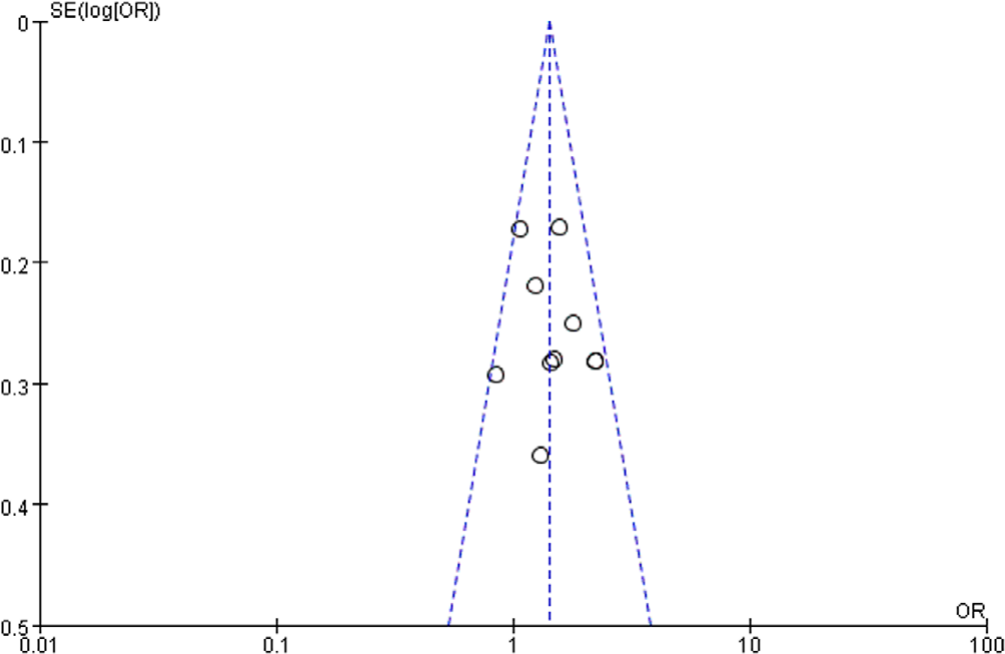
Funnel plot for publication bias of the observational studies that investigated incidence differences of postoperative pneumonia (POP) between males and females after hip fracture surgery

Ten studies^4^, ^5^, ^6^, ^7^, ^8^, ^9^, ^10^, ^11^, ^12^, ^13^ involving 12,084 patients reported the sex for each group. A random effects model was applied because significant heterogeneity was observed among these studies (*P* < 0.0001, *I* ^2^ = 77%). A significant difference was observed in the incidence of POP between different sexes (*OR* = 1.41, 95% *CI*: 1.02–1.93, *P* = 0.04) (Table 2, Fig. 2b). However, the *I* ^2^ value for five HQ studies^5^, ^7^, ^9^, ^11^, ^13^ decreased to 0%, and the significance remained unchanged (*OR* = 1.66, 95% *CI*: 1.30–2.13, *P* < 0.0001), indicating the reliability of the result was (Fig. S1b). The assessment of publication bias using funnel plots revealed that no potential publication bias existed among the included studies (Fig. 3).

### Patient Health Status‐Related Risk Factors

#### ASA classification

Six studies^4^, ^6^, ^7^, ^8^, ^11^, ^13^ involving 8911 patients reported the ASA classification for each group. A random effects model was applied because significant heterogeneity was observed among these studies (*P* < 0.0001, *I* ^2^ = 90%). A significant difference was observed in the incidence of POP between patients with ASA ≥3 and those with ASA <3 (*OR* = 3.84, 95% *CI*: 1.87–6.47, *P* < 0.0001) (Table 2, Fig. 2c). However, after eliminating MQ studies, the *I* ^2^ value for three HQ studies^7^, ^11^, ^13^ decreased to 48%, and the significance remained unchanged (*OR* = 2.97, 95% *CI*: 2.13–4.16, *P* < 0.0001), indicating the reliability of the results (Fig. S1c).

#### Functional Status

Two studies^8^, ^11^ involving 2398 patients reported the functional status for each group. A random effects model was applied because significant heterogeneity was observed among these studies (*P* < 0.0001, *I* ^2^ = 88%). A significant difference was observed in the incidence of POP between patients in the dependent and independent functional status groups (*OR* = 5.23, 95% *CI*: 2.18–12.54, *P* = 0.0002) (Table 2, Fig. 2d). However, only two studies that had positive findings provided information on the patient's functional status. Considering the significant heterogeneity and fewer studies, the evidence required to draw a conclusion was insufficient.

#### History of Smoking

Seven studies^4^, ^7^, ^8^, ^11^, ^12^, ^13^ involving 9621 patients reported a history of smoking for each group. A fixed effects model was applied because no significant heterogeneity was observed among these studies (*P* = 0.06, *I* ^2^ = 50%). A significant difference was observed in the incidence of POP between smokers and non‐smokers (*OR* = 1.33, 95% *CI*: 1.07–1.65, *P* = 0.01) (Table 2, Fig. 2e).

#### COPD

Eight studies involving^4^, ^5^, ^6^, ^8^, ^10^, ^11^, ^12^, ^13^ 7442 patients reported the presence/absence of COPD for each group. A random effects model was applied because significant heterogeneity was observed among these studies (*P* < 0.0001, *I* ^2^ = 86%). A significant difference was observed in the incidence of POP between patients with and without COPD (*OR* = 3.84, 95% *CI*: 1.87–6.47, *P* < 0.0001) (Table 2, Fig. 2f). However, after sensitivity analysis by eliminating studies published after 2020, the *I* ^2^ value for five studies^4^, ^5^, ^8^, ^10^, ^13^ decreased to 0%, and the significance remained unchanged (*OR* = 2.21, 95% *CI*: 1.61–3.02, *P* < 0.0001) (Fig. S1d). Meanwhile, the three excluded studies considered COPD a risk factor for POP, indicating the reliability of the result.

#### Diabetes Mullites (DM)

Ten studies^4^, ^5^, ^6^, ^7^, ^8^, ^9^, ^10^, ^11^, ^12^, ^13^ involving 12,084 patients reported the presence/absence of DM for each group. A fixed effects model was applied because no significant heterogeneity was observed among these studies (*P* = 0.14, *I* ^2^ = 33%). A significant difference was observed in the incidence of POP between patients with and without DM (*OR* = 1.19, 95% *CI*: 1.01–1.40, *P* = 0.04) (Table 2, Fig. 2g).

#### Coronary Heart Disease (CHD)

Seven studies^4^, ^5^, ^6^, ^7^, ^8^, ^9^, ^12^ involving 9960 patients reported the presence/absence of CHD for each group. A random effects model was applied because significant heterogeneity was observed among these studies (*P* = 0.008, *I* ^2^ = 65%). A significant difference was observed in the incidence of POP between patients with and without CHD (*OR* = 1.74, 95% *CI*: 1.23–2.46, *P* = 0.002) (Table 2, Fig. 2h). However, after sensitivity analysis by eliminating two studies with sample sizes <1000,^9^, ^12^ the *I* ^2^ value for the five other studies^4^, ^5^, ^6^, ^7^, ^8^ decreased to 48%, and the significance remained unchanged (*OR* = 2.19, 95% *CI*: 1.75–2.74, *P* < 0.0001), indicating the reliability of the result (Fig. S1e).

#### Arrhythmia

Three studies^4^, ^8^, ^12^ involving 3262 patients reported the presence/absence of arrhythmia for each group. A fixed effects model was applied because no significant heterogeneity was observed among these studies (*P* = 0.29, *I* ^2^ = 20%). A significant difference was detected in the incidence of POP between patients with and without arrhythmia (*OR* = 1.47, 95% *CI*: 1.01–2.14, *P* = 0.04) (Table 2, Fig. 2i).

#### Cerebrovascular Disease (CVA)

Eight studies^4^, ^5^, ^6^, ^7^, ^9^, ^10^, ^12^, ^13^ involving 7686 patients reported the presence/absence of CVA for each group. A fixed effects model was applied because no significant heterogeneity was observed among these studies (*P* = 0.30, *I* ^2^ = 17%). A significant difference was observed in the incidence of POP between patients with and without CVA (*OR* = 1.88, 95% *CI*: 1.56–2.27, *P* < 0.0001) (Table 2, Fig. 2j).

#### Dementia

Four studies^4^, ^10^, ^11^, ^13^ involving 3553 patients reported the presence/absence of dementia for each group. A random effects model was applied because significant heterogeneity was observed among these studies (*P* = 0.008, *I* ^2^ = 82%). A significant difference was observed in the incidence of POP between patients with and without dementia (*OR* = 2.36, 95% *CI*: 1.04–5.36, *P* = 0.04) (Table 2, Fig. 2k). However, after sensitivity analysis by eliminating two studies with sample sizes <500,^10^, ^13^, the *I* ^2^ value for the two other studies^4^, ^11^ decreased to 18%, and the significance remained unchanged (*OR* = 4.71, 95% *CI*: 2.68–8.26, *P* < 0.0001), indicating the reliability of the result (Fig. S1f).

#### Chronic Renal Failure (CRF)

Four studies^4^, ^5^, ^6^, ^7^ involving 7226 patients reported the presence/absence of CRF for each group. A fixed effects model was applied because significant heterogeneity was observed among these studies (*P* = 0.51, *I* ^2^ = 0%). A significant difference was detected in the incidence of POP between patients with and without CRF (*OR* = 1.85, 95% *CI*: 1.29–2.67, *P* = 0.0009) (Table 2, Fig. 2l).

#### Preoperative Cr

Four studies^5^, ^8^, ^9^, ^12^ involving 3889 patients reported the preoperative Cr for each group. A fixed effects model was applied because no significant heterogeneity was observed among these studies (*P* = 0.15, *I* ^2^ = 44%). A significant difference was observed in the preoperative Cr between patients with and without POP (*MD* = 5.32, 95% *CI*: 0.55–10.08, *P* < 0.0001) (Table 2, Fig. 2o).

#### Preoperative Alb

Four studies^4^, ^5^, ^8^, ^12^ involving 4417 patients reported the preoperative Alb for each group. A random effects model was applied because significant heterogeneity was observed among these studies (*P* < 0.0001, *I* ^2^ = 87%). A significant difference was observed in the preoperative Alb between patients with and without POP (*MD* = −3.24, 95% *CI*: −4.59 to −1.89, *P* < 0.0001). A subgroup analysis was attempted based on nationality, age, and fracture type; however, significant heterogeneity was not ruled out. Heterogeneity might be the result of multiple factors. However, the four included studies considered preoperative Alb a risk factor for POP, indicating the reliability of the result (Table 2, Fig. 2p).

#### Preoperative Hypoalbuminemia

Four studies^7^, ^11^, ^12^, ^13^ involving 3932 patients reported preoperative hypoalbuminemia for each group. A random effects model was applied because significant heterogeneity was observed among these studies (*P* < 0.0001, *I* ^2^ = 90%). A significant difference was observed in preoperative hypoalbuminemia between patients with and without POP (*OR* = 3.33, 95% *CI*: 1.32–8.37, *P* = 0.01) (Table 2). After sensitivity analysis by excluding one of the included studies,^13^ the significance changed (*OR* = 2.80, 95% *CI*: 0.89–8.80, *P* = 0.08), indicating that the result was unreliable and this factor might not be suitable for meta‐analysis (Fig. S1i).

### Surgery‐Related Risk Factors

#### Delayed Surgery

When the duration between the time of injury and surgery was >48 h, we referred to it as delayed surgery. Three studies^8^, ^10^, ^13^ involving 1952 patients provided information on whether the surgery was performed within 48 h after injury for each group. A random effects model was applied because significant heterogeneity was observed among these studies (*P* = 0.05, *I* ^2^ = 67%). A significant difference was observed in the incidence of POP between patients undergoing delayed surgery and others (*OR* = 6.40, 95% *CI*: 3.00–13.68, *P* < 0.0001). The three included studies considered delayed surgery a risk factor for POP, indicating the reliability of the result (Table 2, Fig. 2n).

#### Time from Injury to Surgery

Five studies^5^, ^6^, ^7^, ^9^, ^12^ involving 7418 patients reported the time from injury to surgery for each group. A random effects model was applied because significant heterogeneity was observed among these studies (*P* < 0.0001, *I* ^2^ = 89%). A significant difference was observed in the time from injury to surgery between patients with and without POP (*MD* = 2.84, 95% *CI*: 0.94–4.74, *P* = 0.003). After subgroup analysis based on study endpoints, the *I* ^2^ value for two studies^9^, ^12^ that regarded postoperative 30‐day pneumonia as an outcome decreased to 0%, and the significance remained unchanged (*MD* = 5.80, 95% *CI*: 4.31–7.29, *P* < 0.0001). This finding indicated that the time from injury to surgery was a risk factor for postoperative 30‐day pneumonia; however, the evidence was scarce (Fig. S1g). Significant heterogeneity was observed among the three other studies^5^, ^6^, ^7^ that considered discharge as the study endpoint (*P* < 0.02, *I* ^2^ = 74%) and no significant difference was observed (*MD* = 1.17, 95% *CI*: −0.10 to 2.43, *P* = 0.07), indicating pooling results based on this factor is unreliable (Fig. S1h).

#### Surgical Procedure

Seven studies^4^, ^5^, ^6^, ^9^, ^10^, ^11^, ^12^ involving 8794 patients reported the surgical procedure for each group. A fixed effects model was applied because no significant heterogeneity was observed among these studies (*P* = 0.25, *I* ^2^ = 23%). A significant difference was detected in the incidence of POP between patients undergoing hip arthroplasty and internal fixation (*OR* = 1.30, 95% *CI*: 1.08–1.56, *P* = 0.006) (Table 2, Fig. 2m).

## Discussion

POP is associated with unsatisfactory short‐ and long‐term outcomes, including prolonged hospital length of stay, more frequent ICU stays, and a higher rate of sepsis, readmission, and mortality, in elderly patients with hip fractures. Considering these adverse events, identifying the high‐risk population by determining the risk factors for POP to prevent it after surgery is essential. This review aims to summarize and evaluate the findings from previous studies investigating the association between potential risk factors and POP in elderly patients with hip fractures undergoing surgery.

We reviewed 10 studies wherein the incidence of POP ranged from 3.5% to 15.2%. This wide range could be explained by differences in institutions, inclusion and exclusion criteria, sample size, and levels of the study. The pooled analysis confirmed that advanced age, male sex, ASA classification ≥3, dependent functional status, history of smoking, COPD, DM, CHD, arrhythmia, CVA, dementia, CRF, hip arthroplasty, delayed surgery, and preoperative Cr and Alb are significantly associated with POP in elderly patients after hip fracture surgery.

### Demographic Variables‐Related Risk Factors

Reasonable evidence suggested that age was associated with POP in elderly patients with hip fractures. Data from the included studies revealed that the ORs of age were statistically significant. However, age as a risk factor was eliminated in studies^8^, ^9^, ^12^ after multivariate regression analysis, indicating that age is highly collinear with or confounding by other risk factors. This suggests that increased comorbidities and other issues secondary to senescence might be more responsible for POP. Meanwhile, several authors have reported that age‐related comorbidities and not age itself are risk factors for POP in elderly patients undergoing hip fracture surgery.^21^, ^22^ Therefore, advanced age should not be an absolute surgical contraindication.^23^

Reasonable evidence suggested that the male sex was a risk factor for POP in elderly patients with hip fractures. In patients undergoing THA, the male sex had the weakest association with POP.^24^ However, in another study conducted in an elderly cohort with hip fractures, the male sex was the strongest independent risk factor,^25^ indicating that the influence of sex on the morbidity of POP might get stronger with age. Although the incidence of hip fractures in men was lower than that in women, male predilection was observed in elderly individuals aged >80 years, and one‐third of these patients died within a year.^26^ It is believed that elderly males always had a poorer preoperative health status (according to the ASA classification) with more comorbidities,^27^ explaining why the male sex was potentially a risk factor for POP in this meta‐analysis.

### Patient Health Status‐Related Risk Factors

Strong evidence indicated that a history of smoking was a risk factor for POP in elderly patients with hip fractures. Smoking is associated with an increased risk of hip fracture and chronic respiratory disease and decreased pulmonary function. Regional dysfunctional immunity and microbial adhesion caused by smoking could result in pneumonia.^28^ Reportedly, twice the incidence of POP was observed in non‐cardiac surgical patients with a history of smoking.^29^ Smoking‐induced POP accounts for the increased mortality in elderly patients with hip fractures.^30^ Ally et al. identified smoking as a risk factor for POP after THA and knee arthroplasty and reported that smoking cessation at least 4 weeks prior to surgery could markedly reduce the incidence of POP.^31^ However, early surgery is currently recommended in elderly patients with hip fractures, and a prolonged preoperative waiting time is associated with poor prognosis.^32^ The development of perioperative airway management strategies, particularly for patients who smoke, might help lower the incidence of POP; however, further research is warranted.

Retention of airway secretion secondary to COPD could lead to postoperative pulmonary complications and worsen prognosis in elderly patients with hip fractures.^33^ If POP is concomitant with COPD, the mortality rate increases by seven times in geriatric patients with hip fracture.^34^ This meta‐analysis reported that COPD was an important factor in pneumonia risk assessment. However, three studies published after 2020 revealed higher ORs for COPD compared with five other studies, causing significant heterogeneity in the pooled effect. One possible explanation is that modifiable risk factors have been controlled more effectively in recent years by optimizing perioperative management, which makes unmodifiable factors, such as COPD, prominent.

Herein, there was considerable evidence that DM was associated with POP. Patients with DM were susceptible to POP, and the mortality rate further increased if patients with DM were complicated with POP.^35^ Consistent with our results, He et al. reported that trauma patients with DM experienced a higher rate of severe infectious complications, including pneumonia.^36^ With the development of hypoglycaemic agents and emphasis on blood glucose management, most patients with DM are well under control during hospitalization. However, DM was still a strong risk factor for POP, indicating that the influence of DM on a patient's susceptibility to POP might not be limited to hyperglycaemia. As an innate immune‐mediated chronic inflammatory disease, DM induces immune cell dysfunction with abnormal expression of various inflammatory factors *via* several complex signaling pathways.^37^ Further studies are required to identify the association between DM and POP following hip fracture surgery in the elderly.

Herein, CHD and arrhythmia were found to be associated with POP. As reported, POP was associated with cardiovascular diseases regardless of age, sex, and comorbidities.^38^ Patients with CHD or arrhythmia showed decreased activity tolerance and vital capacity, indicating poor pulmonary compliance and self‐purification ability. Moreover, cardiovascular diseases might trigger or aggravate haemodynamic instability and contribute to pulmonary congestion and oedema that could result in an infection.^39^ It was reported that for elderly patients with femoral neck fractures undergoing HHA, CHF doubled the chances of developing POP^40^; however, the findings reported by this meta‐analysis to support the correlation between CHF and POP were insufficient.

Herein, dementia and CVA were found to be associated with POP. In two included studies, dementia was not considered a significant risk factor for POP, causing significant heterogeneity. This could be a result of an inaccurate representation of the population characteristics due to the limited sample size. Dementia‐related dysphagia and decreased muscle mass and connective tissue elasticity in frail senile patients, as well as an incorrect feeding pattern, increased the risk of aspiration, consequently causing pneumonia.^13^, ^41^ Motor, sensory, and cognitive dysfunction secondary to CVA and being long‐term bedridden also contributed to aspiration. Meanwhile, prior stroke could result in abnormal systemic immune responses and increase the patient's susceptibility to POP.^42^ Limited communication and a hidden onset of POP for such patients make early detection challenging and worsen prognosis.^43^

Considerable evidence suggested that CRF was associated with POP in elderly patients after hip fracture surgery. Patients with CRF are immunocompromised and regarded as the most medically vulnerable patients.^44^ Even patients with mild CRF had an extremely high risk of infection, with pulmonary infection accounting for most clinical complications.^45^ One reasonable explanation was that renal insufficiency induces hypoproteinaemia and malnutrition, as well as the accumulation of metabolic products, such as hydrogen ions, histamine, and serotonin, resulting in acidosis and other internal environmental disorders, leading to abnormal humoral cellular immunity.^44^ Consistent with this, strong evidence indicated that an increase in preoperative Cr was a risk factor for POP after hip fracture surgery in elderly patients.

The ASA physical status grading system was developed to evaluate the physical status risk of anesthesia tolerance. ASA classification was the strongest among all potential risk factors for POP after hip fracture surgery individuals with ASA class 4 had a three times higher risk of developing POP compared with ASA class 2 individuals.^46^ Consistent with previous studies, this meta‐analysis also considered ASA class 3 and 4 as important risk factors for POP in elderly patients with hip fractures. Aged patients with a higher ASA classification should be treated as critical individuals and deserve an investment of medical resources to safely go through the high‐risk period after surgery.

Limited evidence existed on the association between dependent functional status and POP. On the one hand, the dependent status of patients is always secondary to other risk factors for POP, such as CVA and dementia. On the other hand, after being in a long‐term dependent functional status, sarcopenia reduces respiratory muscle strength, which is known to regulate effective coughing for clearing the airway,^47^ eventually increasing the risk of POP. Further studies are needed to support this conclusion.

As a marker of malnutrition, hypoalbuminemia was observed in 47% of geriatric patients with hip fractures at admission.^4^, ^48^ Although preoperative albuminemia was significantly associated with POP, we did not draw a conclusion according to this finding owing to the unreliable result in the sensitivity analysis and unexplained high heterogeneity. Serum Alb was associated with collagen synthesis and innate immunity.^49^ Low serum Alb might result in immune dysfunction and lower the patient's ability to fight against infection. Reduction in Alb levels might result in enhanced capillary permeability into the interstitial space,^50^ leading to pleural effusion and pulmonary oedema, augmenting the risk of pneumonia. Sufficient analgesia and anti‐inflammatory agents, prevention of stress ulcers and functional constipation, early rehabilitation of gastrointestinal function, and opportune psychological counseling are effective auxiliary measures to avoid hypoalbuminemia and should be considered for individuals belonging to the POP‐high‐risk cohort.

### Surgery‐Related Risk Factors

This meta‐analysis indicated that delayed surgery and hip arthroplasty were risk factors for POP in elderly patients with hip fractures.

The threshold time to define delayed surgery varied from 6 to 72 h, with 48 h being the most common threshold time. Surgical delays were associated with increased postoperative complications in elderly patients with hip fractures.^51^ A delay of only 12 h increased the risk of pneumonia in patients without any known preoperative comorbidity.^52^ In a previous meta‐analysis on the impact of surgery timing in elderly hip fracture patients, early surgery was associated with fewer perioperative complications, including pneumonia.^53^

Although time from injury to surgery seemed to be associated with POP in this meta‐analysis, the result was unreliable after performing the subgroup analysis. Limited evidence indicated that a prolonged time from injury to surgery increased the risk of postoperative 30‐day pneumonia; however, the effect on the incidence of in‐hospital POP was uncertain as it lacked sufficient evidence. According to this result, the short‐term prognosis of such patients might not be significantly affected by a prolonged preoperative waiting time, while medium‐ and long‐term complications might be affected to a certain degree. There are variable factors of prolonged time from injury to surgery, including organizational reasons, a patient's medical condition, “weekend effect,” etc.^52^ A prolonged waiting time caused by preoperative physiological optimisation signifies a poor physical status and a higher risk of postoperative complications, which might be a reasonable explanation. Further prospective studies are required to investigate whether prolonging the preoperative waiting time or surgical delays could increase the risk of POP after controlling confounding factors, which is vital for the development of decided strategies.

In this meta‐analysis, considerable evidence revealed that compared with internal fixation, elderly patients with hip fractures undergoing hip arthroplasty were more likely to develop POP. It was consistent with the findings of Lv et al. that patients who underwent closed reduction and intramedullary nailing had the lowest risk of POP development compared with other procedures.^4^ Compared with joint arthroplasty, most internal fixations for patients with hip fractures were performed in a minimally invasive manner. Less trauma means less damage and quicker recovery, resulting in lesser postoperative complications.^54^ Regarding the anesthesia type, there was no significant difference in the incidence of POP among the different anesthesia types. This result is consistent with a previous meta‐analysis.^55^ These results suggest that the inescapable impact that surgical trauma had on POP and general anesthesia might not contribute to the development of POP.

### Strengths and Limitations

The present meta‐analysis has several strengths. To our knowledge, this is the first meta‐analysis on risk factors of POP following hip fracture surgery in the specific cohort of elderly. To avoid inaccuracy caused by inconsistency in definition of research end point, this meta‐analysis only included studies in which the outcome was POP for certain and the explicit diagnostic criteria was given. Moreover, sensitivity or subgroup analysis were used to account for heterogeneity greater than 50% and positive pooled effects without rational explanation for unacceptable heterogeneity were regarded unreliable, which increased the preciseness of the conclusion.

Our study has some limitations. Firstly, we must acknowledge that some potentially relevant studies were excluded because of a lack of detailed case definitions. Secondly, we used a random effects model for certain pooled analyses with significant heterogeneity. Third, although heterogeneity diminished after sensitivity or subgroup analyses, we failed to completely understand the source of heterogeneity. When significant heterogeneity could not be explained, or sensitivity analysis rated an inconstant positive result, we employed a conservative approach and refused to draw a conclusion. Finally, for the purpose of the investigation, most relevant studies were designed in a retrospective manner, wherein recall bias could occur easily. Although this meta‐analysis investigates certain risk factors for POP after hip fracture surgery in elderly individuals, these results should be treated cautiously for potential faults owing to the above limitations. Further studies with a prospective design should be conducted to verify our findings.

### Conclusions

The following are the risk factors for POP in geriatric patients with hip fractures: advanced age, male sex, ASA classification ≥3, dependent functional status, history of smoking, COPD, DM, CHD, arrhythmia, CVA, dementia, CRF, hip arthroplasty, delayed surgery, increased preoperative Cr, and decreased preoperative Alb. Early risk assessment and implementation of preventive measures might help lower the incidence of POP and improve a patient's prognosis.

## Authors’ Contributions

YG has contributed to the conception, study design, and drafting of the study. YZ, WG, and LS have contributed to the acquisition, analysis, and interpretation of the data. LS, YZ, YL, HC, and YR have revised the manuscript. All authors have approved the final version of the manuscript and have agreed to be personally accountable for the author's contributions and questions related to the accuracy or integrity of any part of the work.

## Conflicts of Interest

On behalf of all authors, the corresponding author states that there is no conflict of interest.

## Funding

This work was supported by Winfast Charity Foundation (YL20220525).

## Supporting information


**Figure S1.** Forest plots of the meta‐analyses of certain variables after subgroup or sensitivity analyses. **a.** age, **b.** sex, **c**. American Society of Anaesthesiologists classification ≥3, **d**. chronic obstructive pulmonary disease, **e**. coronary heart disease, **f**. dementia, **g**. time from injury to surgery (postoperative 30‐day pneumonia), **h.** time from injury to surgery (postoperative pneumonia before discharge), **i.** preoperative hypoalbuminemia
