# Supplementary material for: What are Risk Factors of Postoperative Pneumonia in Geriatric Individuals after Hip Fracture Surgery: A Systematic Review and Meta‐Analysis
Source: Orthop Surg. 2022 Dec 15;15(1):38–52. doi: 10.1111/os.13631 (PMC9837248; doi:10.1111/os.13631)
Supplement: Supplementary file 1 — Figure S1. Forest plots of the meta‐analyses of certain variables after subgroup or sensitivity analyses. a. age, b. sex, c. American Society of Anaesthesiologists classification ≥3, d. chronic obstructive pulmonary disease, e. coronary heart disease, f. dementia, g. time from injury to surgery (postoperative 30‐day pneumonia), h. time from injury to surgery (postoperative pneumonia before discharge), i. preoperative hypoalbuminemia [file OS-15-38-s001.docx]

**Fig. S1** Forest plots of the meta-analyses of certain variables after subgroup or sensitivity analyses. **a.** age, **b.** sex, **c**. American Society of Anaesthesiologists classification ≥3, **d**. chronic obstructive pulmonary disease, **e**. coronary heart disease, **f**. dementia, **g**. time from injury to surgery (postoperative 30-day pneumonia), **h.** time from injury to surgery (postoperative pneumonia before discharge), **i.** preoperative hypoalbuminemia.

**a.**

**
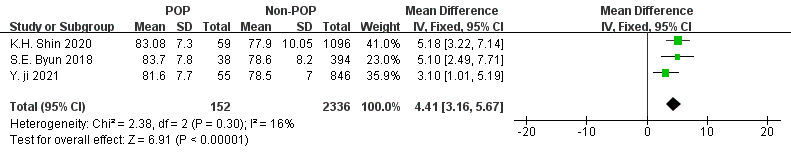
**

**b.**

**
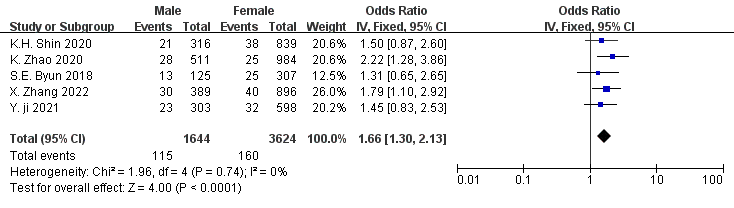
**

**c.
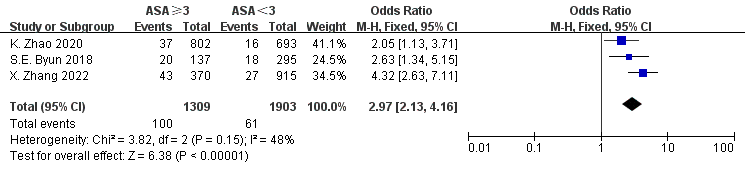
**

**d.
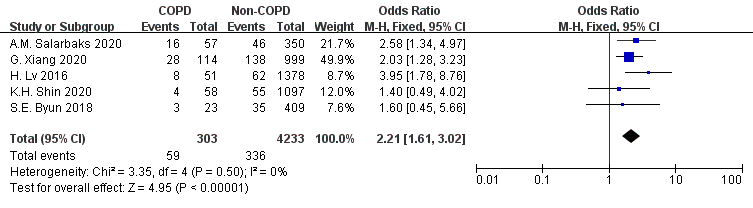
**

**e.**

**
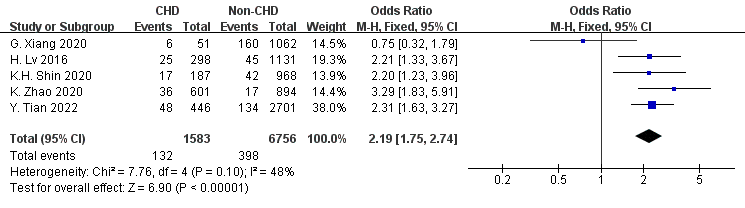
**

**f.
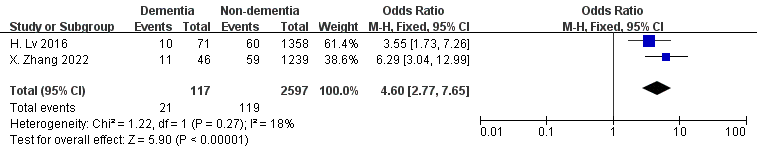
**

**g.**

**
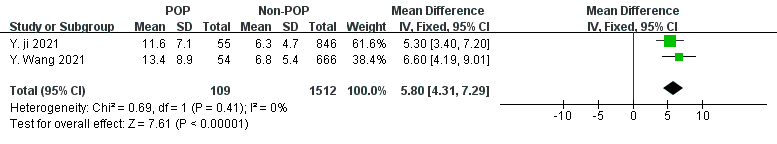
**

**h.
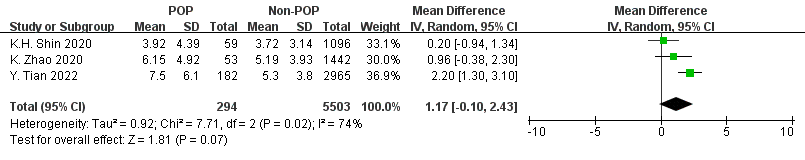
**

**i.
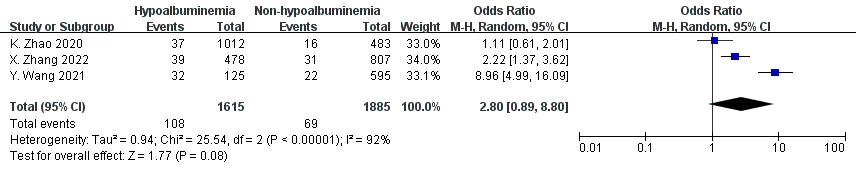
**
